# Supplementary material for: Limitations of PICADAR as a diagnostic predictive tool for primary ciliary dyskinesia
Source: Front Mol Biosci. 2025 Dec 8;12:1691758. doi: 10.3389/fmolb.2025.1691758 (PMC12722925; doi:10.3389/fmolb.2025.1691758)
Supplement: Supplementary file 1 [file Table1.docx]

**Supplementary table 1: Affected genes in mutant PCD individuals**

*Genes that are associated with pathognomonic ciliary ultrastructure defects detectable by transmission electron microscopy (hallmark defects) according to Shoemark *et al.* (Shoemark et al., 2020).

| **Affected genes** | **Number of PCD individuals** |
| --- | --- |
| *CCDC103** | 6 |
| *CCDC39** | 7 |
| *CCDC40** | 27 |
| *CCNO* | 1 |
| *CFAP221* | 1 |
| *CFAP298** | 2 |
| *CFAP300** | 3 |
| *CFAP45* | 1 |
| *CFAP46* | 1 |
| *CFAP54* | 1 |
| *CFAP74* | 1 |
| *DNAAF1** | 3 |
| *DNAAF2** | 3 |
| *DNAAF3** | 6 |
| *DNAAF4** | 3 |
| *DNAAF5** | 1 |
| *DNAAF6** | 5 |
| *DNAAF11** | 3 |
| *DNAH11* | 32 |
| *DNAH5** | 46 |
| *DNAH9** | 2 |
| *DNAI1** | 26 |
| *DNAI2** | 4 |
| *DRC1* | 2 |
| *DRC2* | 3 |
| *FOXJ1* | 2 |
| *GAS8* | 1 |
| *HYDIN* | 13 |
| *IFT74* | 2 |
| *NEK10* | 4 |
| *ODAD1** | 7 |
| *ODAD2** | 6 |
| *ODAD3** | 3 |
| *ODAD4** | 4 |
| *OFD1* | 3 |
| *RPGR* | 1 |
| *RSPH1* | 5 |
| *RSPH4A* | 5 |
| *RSPH9* | 5 |
| *SPAG1** | 4 |
| *SPEF2* | 3 |
| *ZMYND10** | 11 |
